# Supplementary material for: Quantitating primer-template interactions using deconstructed PCR
Source: PeerJ. 2024 Aug 8;12:e17787. doi: 10.7717/peerj.17787 (PMC11317036; doi:10.7717/peerj.17787)

Figures S1-S16: Template and primer utilization profiles for the initial 16 individual experiments conducted in this study. For each study, varying number of primers and templates were used, as described in Table 1 and Supplemental Table 7. Original experimental names have been retained in these figures. For mMDS plots, samples were color coded by amplification method and different annealing temperatures indicated by shape. Ellipses represent a 95% confidence interval around the centroid. ANOVA was performed to measure differences in measured values by annealing temperature. Intensity scales vary between experiments. All samples were rarefied to 7,000 sequences. Heatmaps are the average of 7-8 technical replicates per condition; all replicates are shown in mMDS plots. (A) For each experiment, primer utilization profiles (PUPs) were generated (left side), and data are presented as mMDS plots (top) and as clustered heatmaps (bottom). Analysis of similarity (ANOSIM) was performed to determine if PUPs were significantly different between TAS and DePCR, regardless of annealing temperature, and within method across annealing temperature. Each slide contains a table showing the percentage of reads with 0, 1, 2 and 3 mismatches between primers and templates, as indicated in experiments with DePCR amplifications. For primer-template interactions with only a single mismatch, percentage of reads with 3' (-2), middle (-8) and 5' (-14) mismatches are shown. The average theoretical melting temperature of primers used in each study are shown. (B) Template profiling analyses were performed (right side), and data are presented as mMDS plots (top) and as clustered heatmaps (bottom). In addition to analysis of sequence data, the expected distribution of reads is shown in orange, both in the mMDS plots and in the heatmap. ANOSIM was performed to determine if template profiles were significantly different between TAS and DePCR, regardless of annealing temperature, and within method across annealing temperature. Ideal scores, as described in text, were calculated to determine which method and annealing temperature generated the closest approximation of the expected template distribution.

Fig. S1

Primer Utilizing Profiles

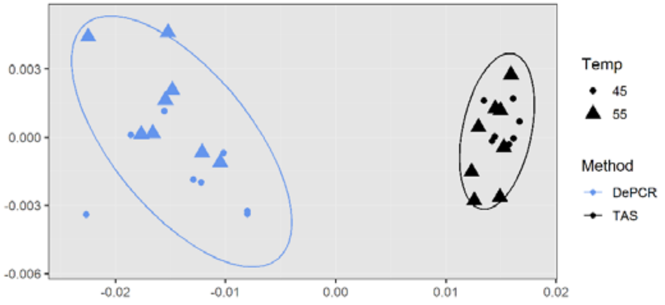

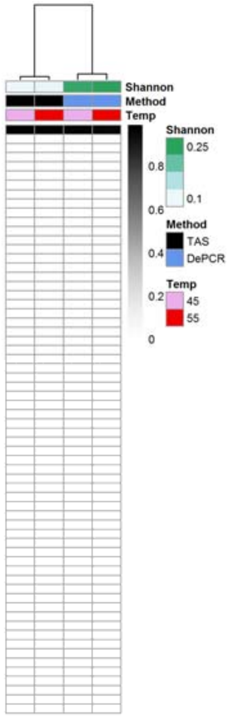

ANOSIM

DePCR vs TAS:
R=1, P=0.0001

TAS45 vs TAS55:
R=0.12; p=0.0012

DePCR45 vs DePCR55:
R=0.099, P=0.109

Comparison	DePCR45	DePCR55	ANOVA
% Reads 0 MM	96.07	95.73	0.07
% Reads 1 MM	3.68	3.99	0.06
% Reads 2 MM	0.25	0.27	0.62
% Reads 3MM	0.00	0.01	0.28
% Reads with 3' MM	26.81	25.29	0.23
% Reads with Mid' MM	30.87	34.19	0.01
% Reads with 5' MM	42.32	40.51	0.17
Average Primer Tm	57.42	57.43	0.02
Average Shannon	0.26	0.27	0.41

Experiment: A1
Primers: 1
Templates: 1

Template Profiles

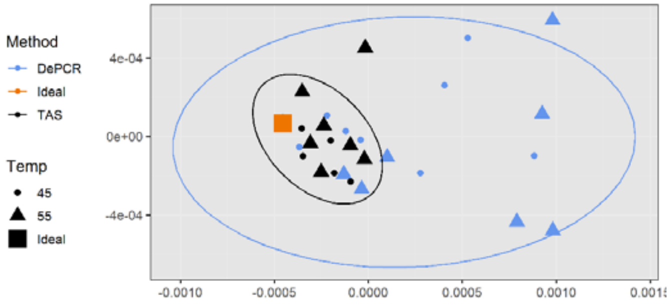

ANOSIM

DePCR vs TAS:
R=0.201, P=0.0003

TAS45 vs TAS55:
R=-0.049; p=0.762

DePCR45 vs DePCR55:
R=-0.015, P=0.478

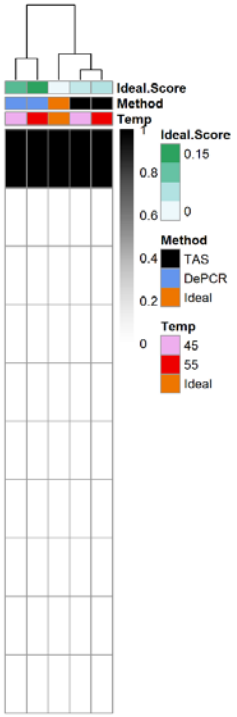

	Comparison	Average Ideal Score	ANOVA
1	TAS	0.06	0.00
	DePCR	0.17	
2	TAS45	0.05	0.39
	TAS55	0.06	
3	DePCR45	0.15	0.38
	DePCR55	0.20	

Fig. S2

Primer Utilizing Profiles

Experiment: A2
Primers: 10
Templates: 1

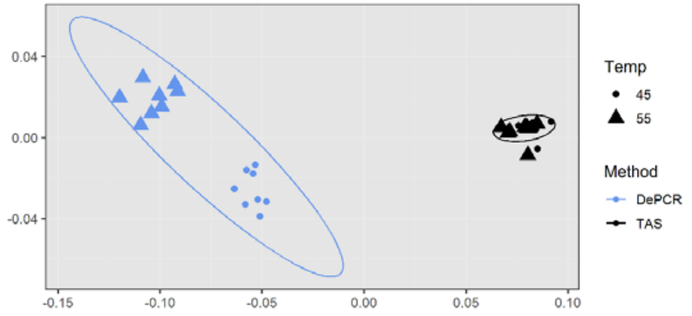

ANOSIM

DePCR vs TAS:
R=1, P=0.0001

TAS45 vs TAS55:
R=0.425; p=0.0009

DePCR45 vs DePCR55:
R= 1, P=0.0002

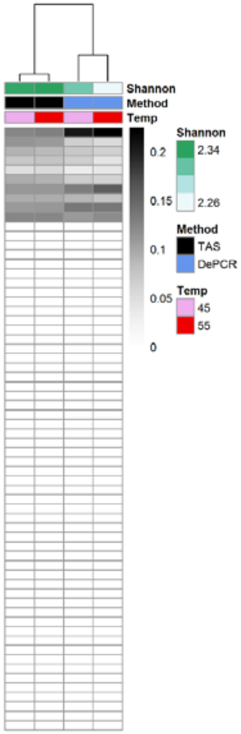

Comparison	DePCR45	DePCR55	ANOVA
% Reads 0 MM	20.51	22.40	0.00
% Reads 1 MM	77.21	75.57	0.00
% Reads 2 MM	2.21	1.99	0.03
% Reads 3MM	0.06	0.04	0.05
% Reads with 3' MM	22.46	19.63	0.00
% Reads with Mid' MM	34.31	36.69	0.00
% Reads with 5' MM	43.23	43.68	0.28
Average Primer Tm	57.79	57.84	0.00
Average Shannon	2.31	2.26	0.00

Template Profiles

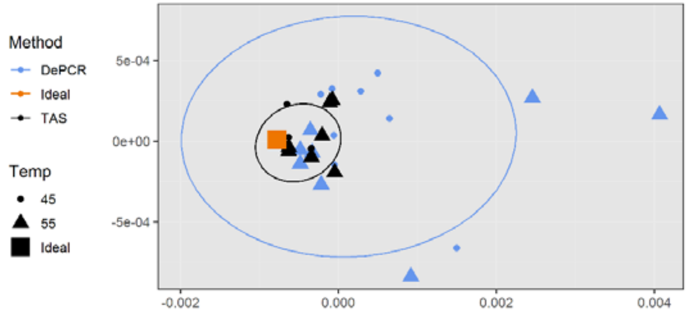

ANOSIM

DePCR vs TAS:
R=0.169, P=0.0003

TAS45 vs TAS55:
R=0.183; p=.031

DePCR45 vs DePCR55:
R=0.063, P=0.00063

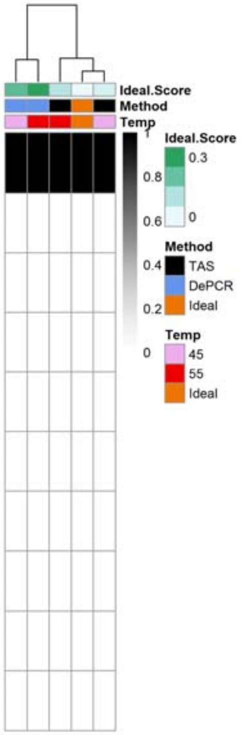

	Comparison	Average Ideal Score	ANOVA
1	TAS	0.06	0.00
	DePCR	0.26	
2	TAS45	0.04	0.04
	TAS55	0.09	
3	DePCR45	0.22	0.53
	DePCR55	0.30	

Fig. S3

Primer Utilizing Profiles

<u>Experiment</u>: A3
Primers: 9
Templates: 1

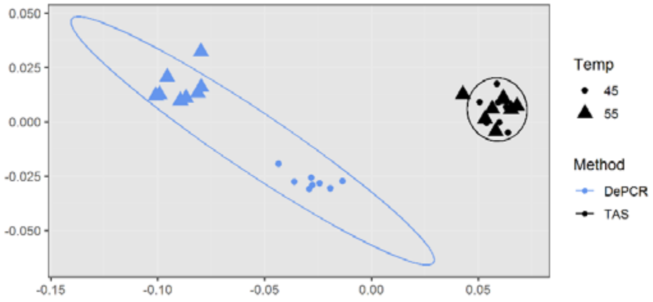

ANOSIM

<u>DePCR vs TAS:</u>
R=0.968, P=0.0001

<u>TAS45 vs TAS55:</u>
R=0.207; p=0.02

<u>DePCR45 vs DePCR55:</u>
R=1, P=0.0002

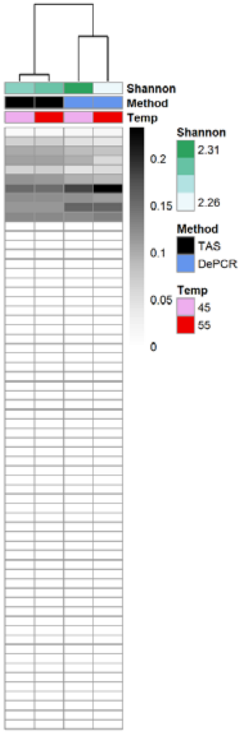

Comparison	DePCR45	DePCR55	ANOVA
% Reads 0 MM	1.39	1.34	0.50
% Reads 1 MM	95.79	95.93	0.28
% Reads 2 MM	2.76	2.66	0.43
% Reads 3MM	0.06	0.07	0.59
% Reads with 3' MM	24.06	20.38	0.00
% Reads with Mid' MM	33.92	37.58	0.00
% Reads with 5' MM	42.03	42.04	0.98
Average Primer Tm	57.81	57.95	0.00
Average Shannon	2.31	2.25	0.00

Template Profiles

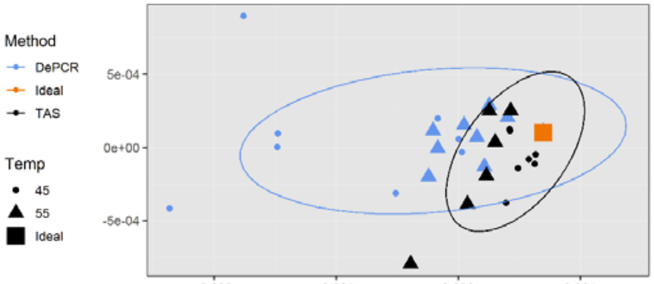

ANOSIM

<u>DePCR vs TAS:</u>
R=0.25, P=0.0001

<u>TAS45 vs TAS55:</u>
R=0.064; p=0.137

<u>DePCR45 vs DePCR55:</u>
R=0.26, P=0.006

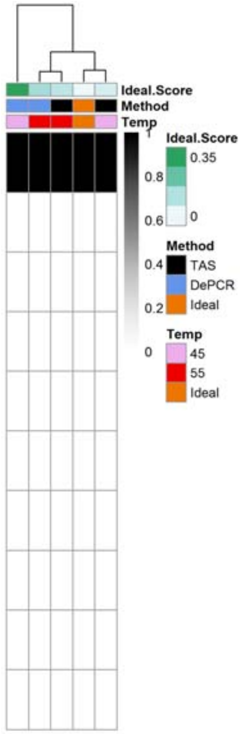

	Comparison	Average Ideal Score	ANOVA
1	TAS	0.08	0.00
	DePCR	0.25	
2	TAS45	0.05	0.17
	TAS55	0.10	
3	DePCR45	0.36	0.00
	DePCR55	0.14	

Fig. S4

Primer Utilizing Profiles

Experiment: A4
Primers: 27
Templates: 1

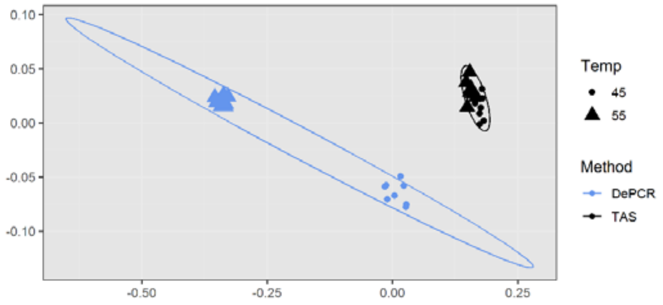

ANOSIM

DePCR vs TAS:
R=0.733, P=000.01

TAS45 vs TAS55:
R=0.894; p=0.0002

DePCR45 vs DePCR55:
R=1, P=0.0002

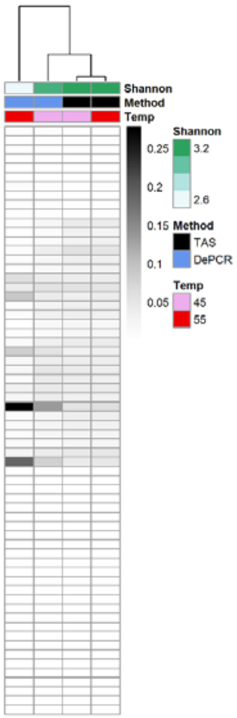

Comparison	DePCR45	DePCR55	ANOVA
**% Reads 0 MM**	0.48	0.39	0.12
**% Reads 1 MM**	2.07	2.45	0.01
**% Reads 2 MM**	96.12	95.82	0.04
**% Reads 3MM**	1.34	1.34	0.95
**% Reads with 3' MM**	25.84	17.10	0.00
**% Reads with Mid' MM**	37.01	44.30	0.00
**% Reads with 5' MM**	37.15	38.59	0.00
**Average Primer Tm**	58.56	58.97	0.00
**Average Shannon**	3.25	2.58	0.00

Template Profiles

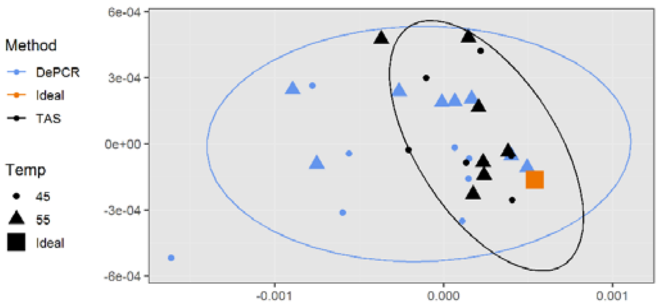

ANOSIM

DePCR vs TAS:
R=0.108, P=0.013

TAS45 vs TAS55:
R=-0.048; p=0.71

DePCR45 vs DePCR55:
R=-0.01, P=0.469

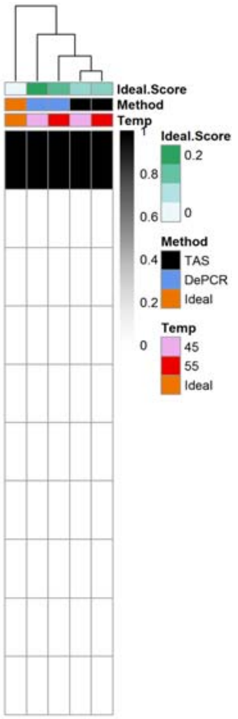

	Comparison	Average Ideal Score	ANOVA
**1**	TAS	0.10	0.02
	DePCR	0.18	
**2**	TAS45	0.09	0.71
	TAS55	0.11	
**3**	DePCR45	0.21	0.37
	DePCR55	0.16	

Fig. S5

Primer Utilizing Profiles

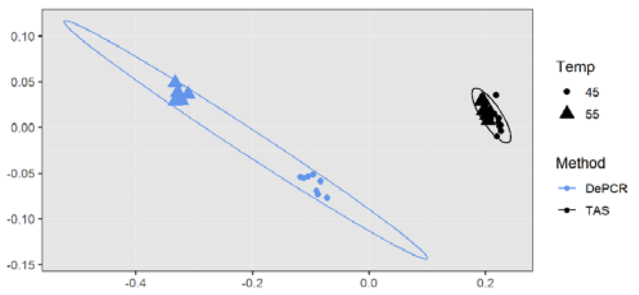

ANOSIM

DePCR vs TAS:
R=1, P=0.0001

TAS45 vs TAS55:
R=1; p=0.0002

DePCR45 vs DePCR55:
R=1, P=0.0002

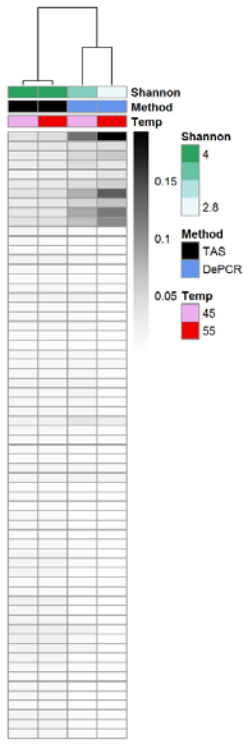

Comparison	DePCR45	DePCR55	ANOVA
% Reads 0 MM	12.16	19.31	0.00
% Reads 1 MM	47.68	65.16	0.00
% Reads 2 MM	29.86	12.81	0.00
% Reads 3MM	10.31	2.72	0.00
% Reads with 3' MM	27.41	21.24	0.00
% Reads with Mid' MM	34.60	37.10	0.00
% Reads with 5' MM	37.99	41.66	0.00
Average Primer Tm	58.18	57.99	0.00
Average Shannon	3.43	2.76	0.00

Experiment: A6
Primers: 64
Templates: 1

Template Profiles

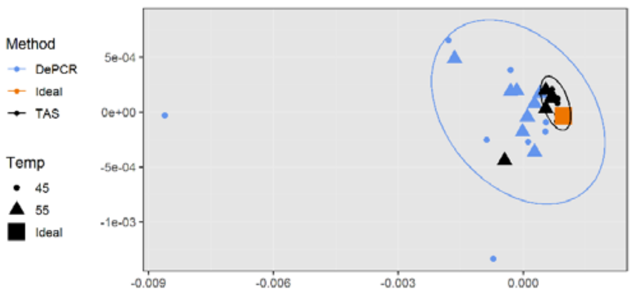

ANOSIM

DePCR vs TAS:
R=0.168, P=0.0002

TAS45 vs TAS55:
R=0.074; p=0.109

DePCR45 vs DePCR55:
R=-0.007, P=0.469

	Comparison	Average Ideal Score	ANOVA
1	TAS	0.05	0.01
	DePCR	0.36	
2	TAS45	0.04	0.31
	TAS55	0.07	
3	DePCR45	0.49	0.25
	DePCR55	0.22	

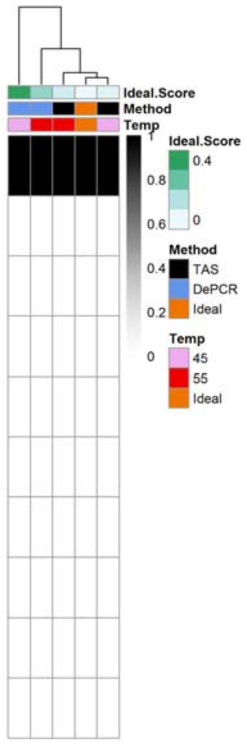

Fig. S6

Primer Utilizing Profiles

Experiment: B1
Primers: 1
Templates: 10

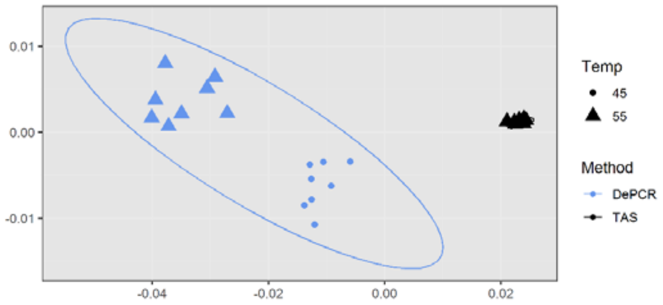

ANOSIM

DePCR vs TAS:
R=0.987, P=0.0001

TAS45 vs TAS55:
R=-0.108; p=0.934

DePCR45 vs DePCR55:
R=1, P=0.0002

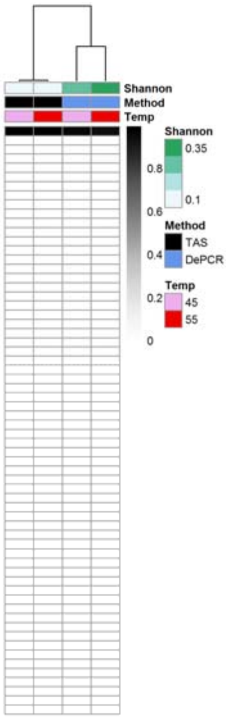

Comparison	DePCR45	DePCR55	ANOVA
% Reads 0 MM	14.27	16.77	0.00
% Reads 1 MM	84.21	81.72	0.00
% Reads 2 MM	1.50	1.48	0.79
% Reads 3MM	0.02	0.03	0.36
% Reads with 3' MM	22.00	19.63	0.00
% Reads with Mid' MM	36.82	39.84	0.00
% Reads with 5' MM	41.18	40.52	0.02
Average Primer Tm	57.42	57.43	0.01
Average Shannon	0.27	0.36	0.00

Template Profiles

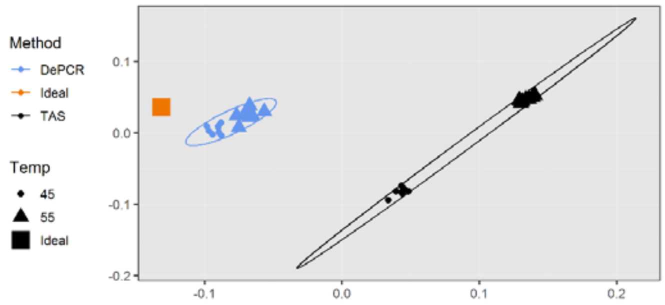

ANOSIM

DePCR vs TAS:
R=0.878, P=0.0001

TAS45 vs TAS55:
R=1; p=0.0002

DePCR45 vs DePCR55:
R=0.909, P=0.0002

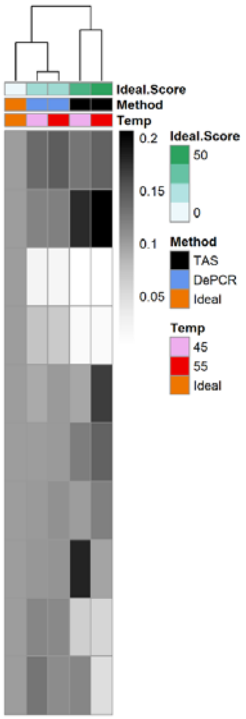

	Comparison	Average Ideal Score	ANOVA
1	TAS	50.80	0.00
	DePCR	22.82	
2	TAS45	45.63	0.00
	TAS55	55.97	
3	DePCR45	22.42	0.12
	DePCR55	23.23	

Fig. S7

Primer Utilizing Profiles

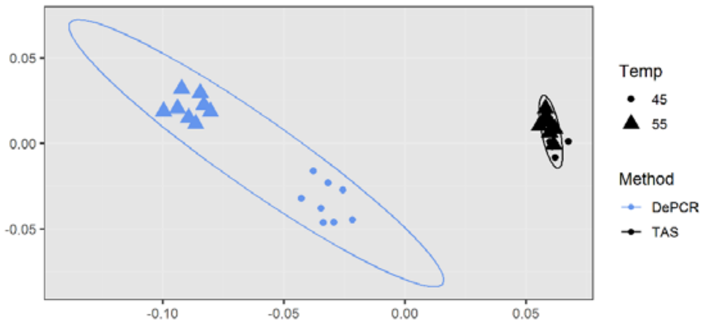

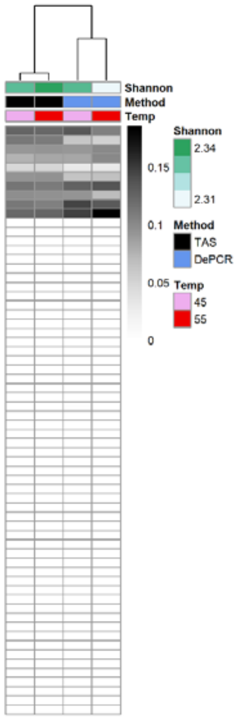

ANOSIM

DePCR vs TAS:
R=0.986, P=0.0001

TAS45 vs TAS55:
R=0.422; p=0.0002

DePCR45 vs DePCR55:
R=1, P=0.0002

Comparison	DePCR45	DePCR55	ANOVA
% Reads 0 MM	28.98	48.05	0.00
% Reads 1 MM	46.54	43.24	0.00
% Reads 2 MM	24.26	8.63	0.00
% Reads 3MM	0.22	0.08	0.00
% Reads with 3' MM	23.28	25.47	0.00
% Reads with Mid' MM	34.68	35.29	0.10
% Reads with 5' MM	42.04	39.24	0.00
Average Primer Tm	57.78	57.87	0.00
Average Shannon	2.33	2.31	0.00

Experiment: B2
Primers: 10
Templates: 10

Template Profiles

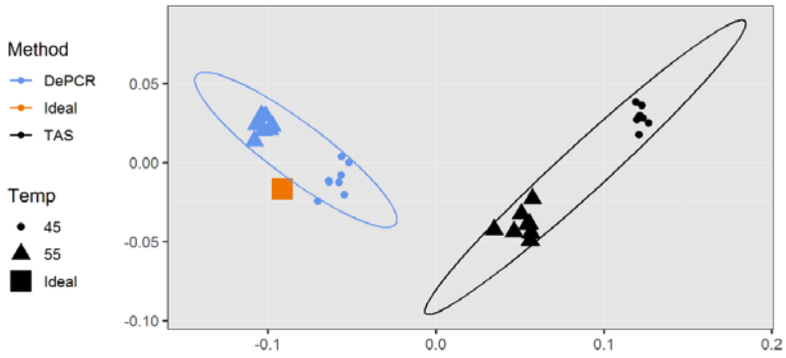

ANOSIM

DePCR vs TAS:
R=0.993, P=0.0001

TAS45 vs TAS55:
R=1; p=0.0002

DePCR45 vs DePCR55:
R=1, P=0.0002

	Comparison	Average Ideal Score	ANOVA
1	TAS	38.60	0.00
	DePCR	16.55	
2	TAS45	45.48	0.00
	TAS55	31.73	
3	DePCR45	18.00	0.00
	DePCR55	15.10	

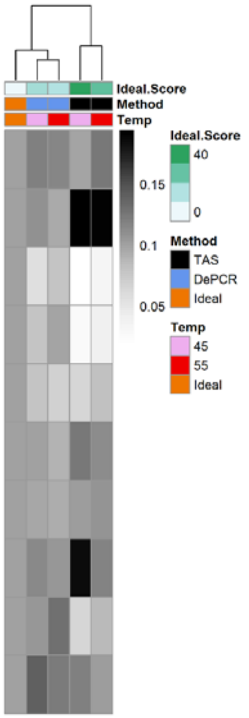

Fig. S8

Primer Utilizing Profiles

Experiment: B3
Primers: 9
Templates: 10

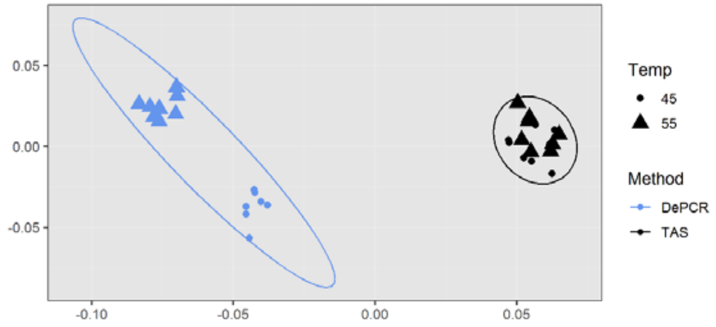

ANOSIM

DePCR vs TAS:
R=1, P=0.0001

TAS45 vs TAS55:
R=-0.051; p=0.671

DePCR45 vs DePCR55:
R=1, P=0.0002

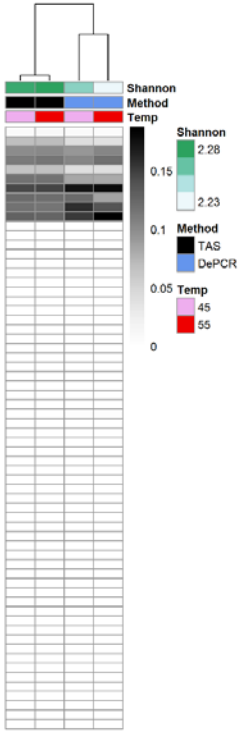

Comparison	DePCR45	DePCR55	ANOVA
**% Reads 0 MM**	29.10	49.31	0.00
**% Reads 1 MM**	42.17	40.86	0.00
**% Reads 2 MM**	28.46	9.73	0.00
**% Reads 3MM**	0.27	0.10	0.00
**% Reads with 3' MM**	23.19	24.82	0.00
**% Reads with Mid' MM**	35.92	36.26	0.40
**% Reads with 5' MM**	40.89	38.91	0.00
**Average Primer Tm**	57.77	57.91	0.00
**Average Shannon**	2.26	2.23	0.00

Template Profiles

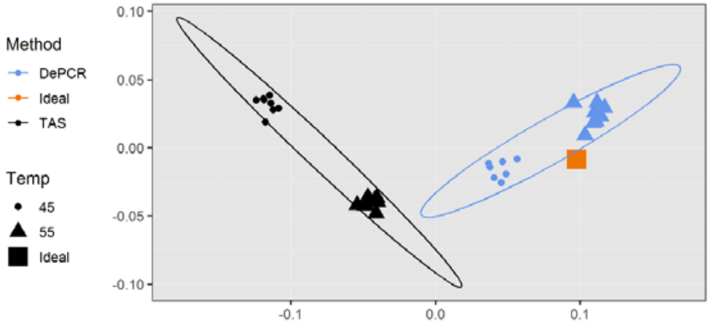

ANOSIM

DePCR vs TAS:
R=0.916, P=0.0001

TAS45 vs TAS55:
R=1; p=0.0002

DePCR45 vs DePCR55:
R=1, P=0.0002

	Comparison	Average Ideal Score	ANOVA
1	TAS	38.38	0.00
	DePCR	16.69	
2	TAS45	45.24	0.00
	TAS55	31.52	
3	DePCR45	19.30	0.00
	DePCR55	14.41	

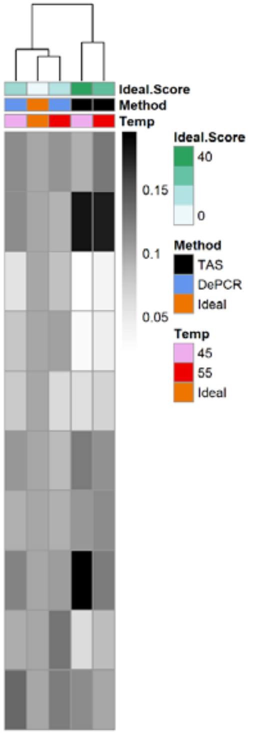

Fig. S9

Primer Utilizing Profiles

Experiment: B4
Primers: 27
Templates: 10

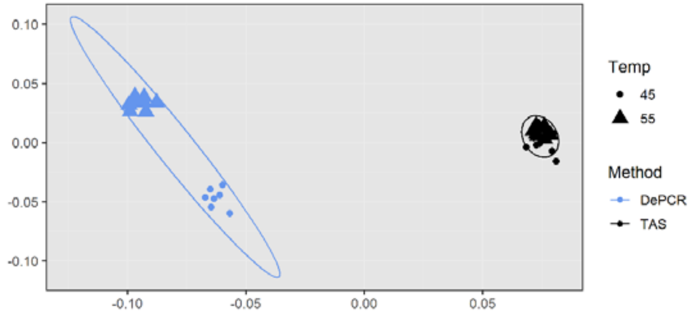

ANOSIM

DePCR vs TAS:
R=1, P=0.0001

TAS45 vs TAS55:
R=0.47; p=0.0002

DePCR45 vs DePCR55:
R=1, P=0.0002

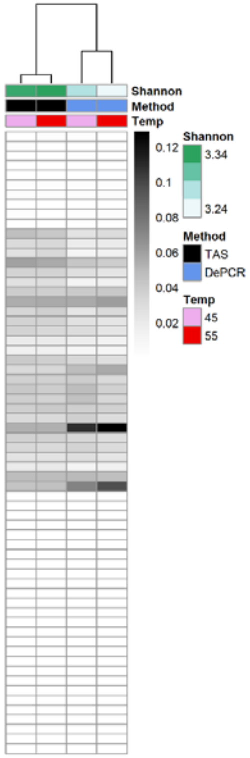

Comparison	DePCR45	DePCR55	ANOVA
% Reads 0 MM	0.56	0.76	0.00
% Reads 1 MM	49.53	74.13	0.00
% Reads 2 MM	45.13	22.37	0.00
% Reads 3MM	4.77	2.75	0.00
% Reads with 3' MM	26.39	22.68	0.00
% Reads with Mid' MM	36.84	40.90	0.00
% Reads with 5' MM	36.77	36.42	0.07
Average Primer Tm	58.59	58.76	0.00
Average Shannon	3.27	3.22	0.00

Template Profiles

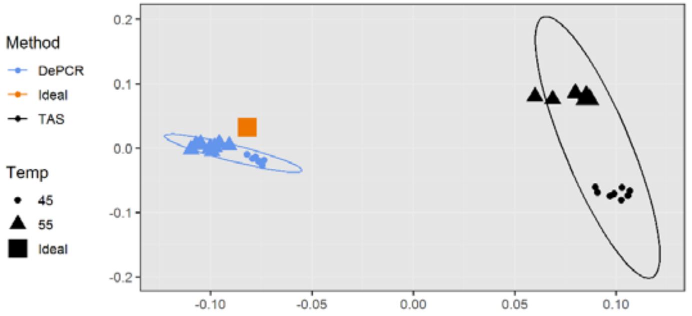

ANOSIM

DePCR vs TAS:
R=1, P=0.0001

TAS45 vs TAS55:
R=1; p=0.0002

DePCR45 vs DePCR55:
R=1, P=0.0002

	Comparison	Average Ideal Score	ANOVA
1	TAS	38.13	0.00
	DePCR	11.53	
2	TAS45	42.14	0.00
	TAS55	34.13	
3	DePCR45	11.82	0.41
	DePCR55	11.28	

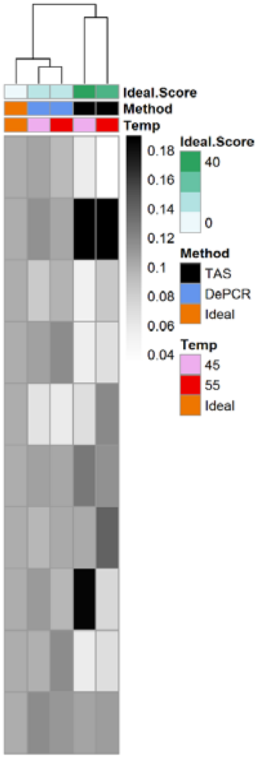

Fig. S10

Primer Utilizing Profiles

Experiment: C1
Primers: 1
Templates: 10

Template Profiles

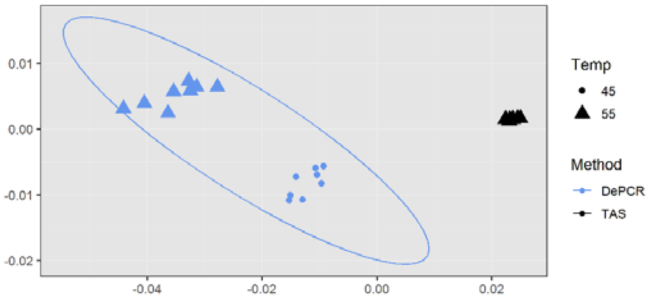

ANOSIM

DePCR vs TAS:
R=0.992, P=0.0001

TAS45 vs TAS55:
R=0.104; p=0.117

DePCR45 vs DePCR55:
R=1, P=0.0002

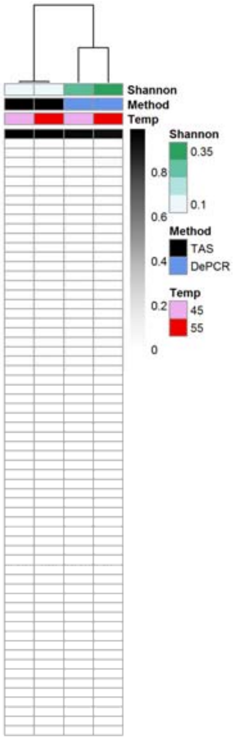

Comparison	DePCR45	DePCR55	ANOVA
% Reads 0 MM	3.42	5.77	0.00
% Reads 1 MM	94.72	92.70	0.00
% Reads 2 MM	1.83	1.51	0.00
% Reads 3MM	0.02	0.02	0.35
% Reads with 3' MM	22.75	20.24	0.00
% Reads with Mid' MM	36.06	39.65	0.00
% Reads with 5' MM	41.19	40.11	0.02
Average Primer Tm	57.42	57.43	0.16
Average Shannon	0.28	0.36	0.00

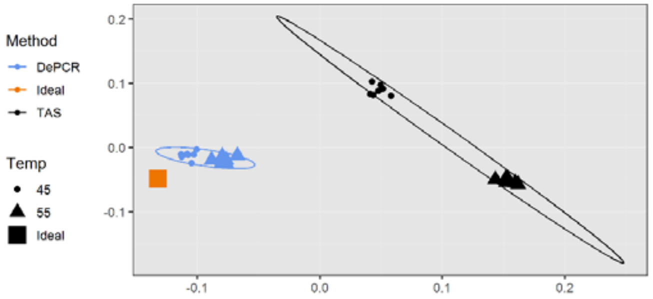

ANOSIM

DePCR vs TAS:
R=0.875, P=0.0001

TAS45 vs TAS55:
R=1; p=0.0002

DePCR45 vs DePCR55:
R=0.993, P=0.0002

	Comparison	Average Ideal Score	ANOVA
1	TAS	54.32	0.00
	DePCR	22.45	
2	TAS45	48.72	0.00
	TAS55	59.91	
3	DePCR45	21.04	0.00
	DePCR55	23.87	

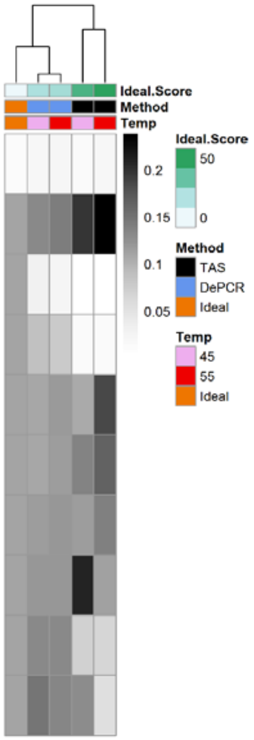

Fig. S11

Primer Utilizing Profiles

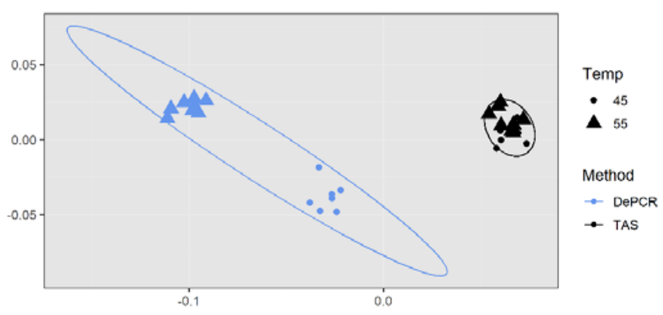

ANOSIM

DePCR vs TAS:
R=0.956, P=0.0001

TAS45 vs TAS55:
R=0.335; p=0.002

DePCR45 vs DePCR55:
R=1, P=0.0002

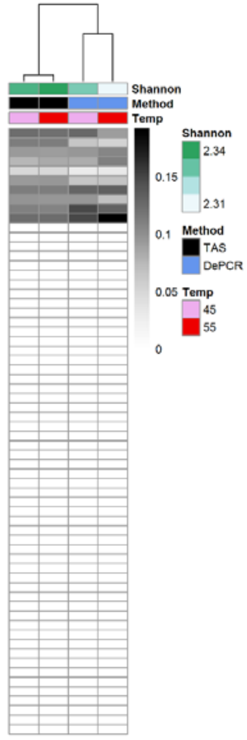

Comparison	DePCR45	DePCR55	ANOVA
% Reads 0 MM	29.58	51.32	0.00
% Reads 1 MM	42.98	38.78	0.00
% Reads 2 MM	27.22	9.83	0.00
% Reads 3MM	0.23	0.07	0.00
% Reads with 3' MM	23.43	26.64	0.00
% Reads with Mid' MM	34.80	34.94	0.65
% Reads with 5' MM	41.77	38.42	0.00
Average Primer Tm	57.79	57.89	0.00
Average Shannon	2.33	2.30	0.00

Experiment: C2
Primers: 10
Templates: 10

Template Profiles

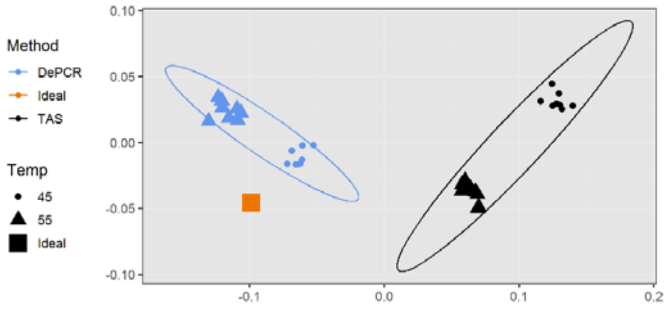

ANOSIM

DePCR vs TAS:
R=1, P=0.0001

TAS45 vs TAS55:
R=1; p=0.0002

DePCR45 vs DePCR55:
R=1, P=0.0002

	Comparison	Average Ideal Score	ANOVA
1	TAS	41.48	0.00
	DePCR	17.56	
2	TAS45	49.17	0.00
	TAS55	33.79	
3	DePCR45	19.07	0.00
	DePCR55	16.04	

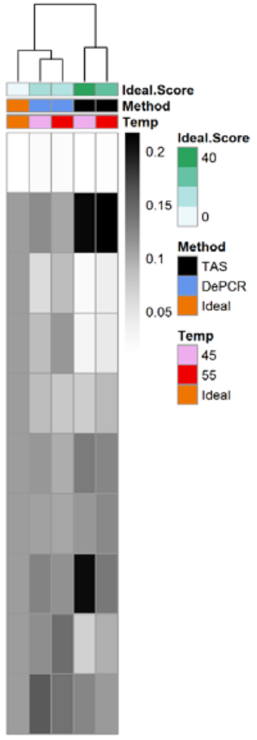

Fig. S12

Primer Utilizing Profiles

Experiment: C3
Primers: 9
Templates: 10

Template Profiles

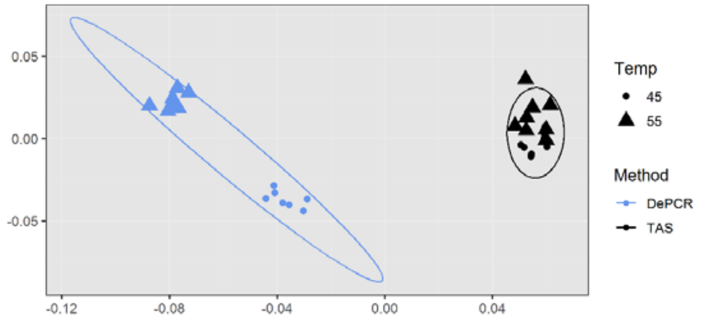

ANOSIM

DePCR vs TAS:
R=1, P=0.0001

TAS45 vs TAS55:
R=0.328; p=0.002

DePCR45 vs DePCR55:
R=1, P=0.0002

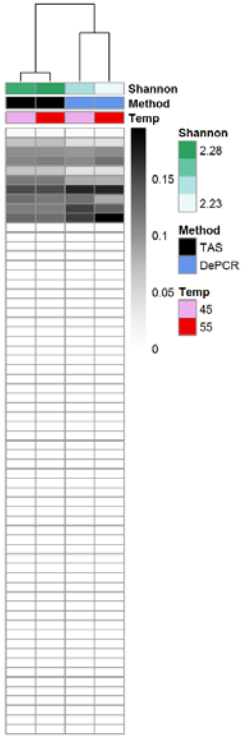

Comparison	DePCR45	DePCR55	ANOVA
% Reads 0 MM	31.68	55.21	0.00
% Reads 1 MM	35.80	34.32	0.00
% Reads 2 MM	32.24	10.39	0.00
% Reads 3MM	0.28	0.09	0.00
% Reads with 3' MM	23.20	25.56	0.00
% Reads with Mid' MM	35.69	36.68	0.03
% Reads with 5' MM	41.11	37.77	0.00
Average Primer Tm	57.75	57.93	0.00
Average Shannon	2.25	2.22	0.00

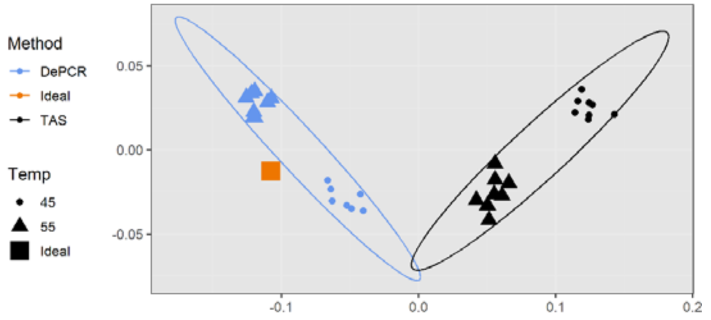

ANOSIM

DePCR vs TAS:
R=0.991, P=0.0001

TAS45 vs TAS55:
R=1; p=0.0002

DePCR45 vs DePCR55:
R=1, P=0.0002

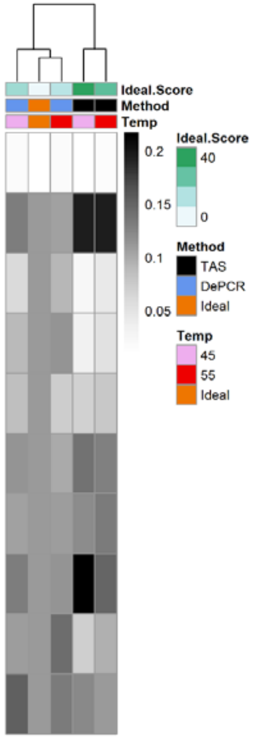

	Comparison	Average Ideal Score	ANOVA
1	TAS	41.53	0.00
	DePCR	17.05	
2	TAS45	48.81	0.00
	TAS55	34.24	
3	DePCR45	20.19	0.00
	DePCR55	14.31	

Fig. S13

Primer Utilizing Profiles

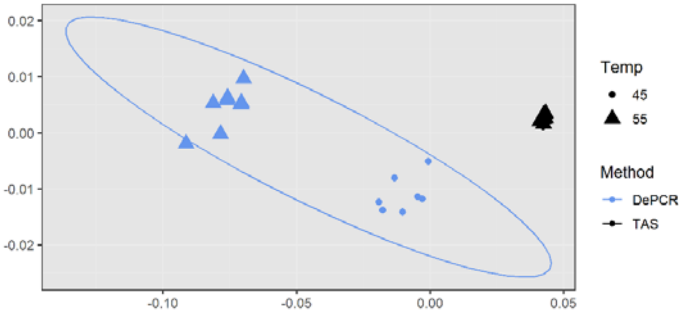

ANOSIM

DePCR vs TAS:
R=0.791, P=0.0001

TAS45 vs TAS55:
R=0.009; p=0.399

DePCR45 vs DePCR55:
R=1, P=0.002

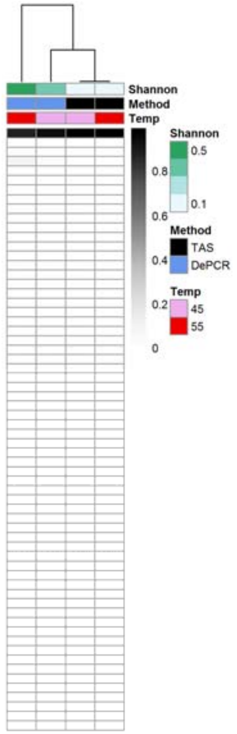

Comparison	DePCR45	DePCR55	ANOVA
**% Reads 0 MM**	13.18	19.78	0.00
**% Reads 1 MM**	85.31	78.86	0.00
**% Reads 2 MM**	1.47	1.34	0.08
**% Reads 3MM**	0.03	0.02	0.13
**% Reads with 3' MM**	97.79	97.65	0.30
**% Reads with Mid' MM**	0.94	0.98	0.66
**% Reads with 5' MM**	1.27	1.37	0.21
**Average Primer Tm**	57.43	57.43	0.00
**Average Shannon**	0.34	0.53	0.00

Experiment: D1
Primers: 1
Templates: 4

Template Profiles

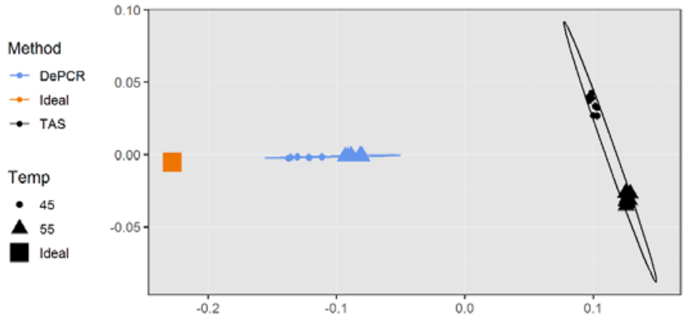

ANOSIM

DePCR vs TAS:
R=1, P=0.0001

TAS45 vs TAS55:
R=1; p=0.0002

DePCR45 vs DePCR55:
R=0.996, P=0.0002

	Comparison	Average Ideal Score	ANOVA
1	TAS	68.46	0.00
	DePCR	24.27	
2	TAS45	65.95	0.00
	TAS55	70.96	
3	DePCR45	20.15	0.00
	DePCR55	27.87	

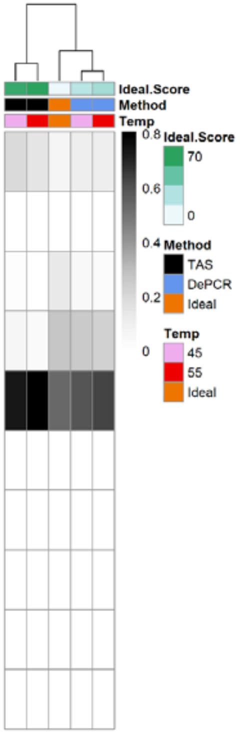

Fig. S14

Primer Utilizing Profiles

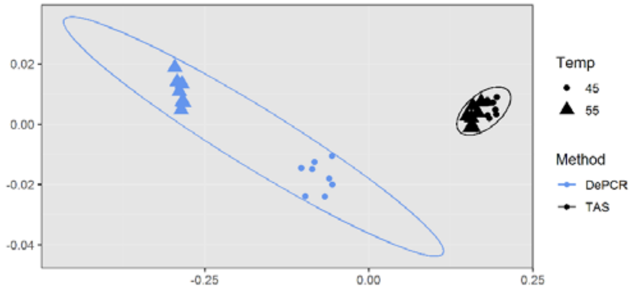

ANOSIM

DePCR vs TAS:
R=0.97, P=0.0001

TAS45 vs TAS55:
R=0.866; p=0.0002

DePCR45 vs DePCR55:
R=1, P=0.0002

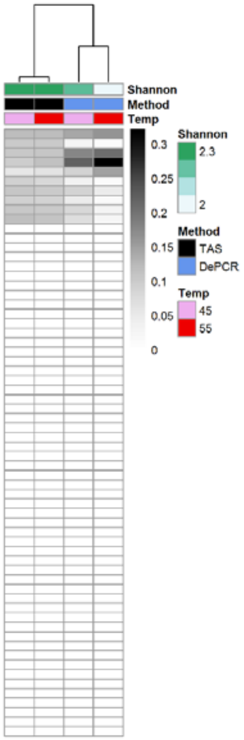

Comparison	DePCR45	DePCR55	ANOVA
% Reads 0 MM	26.59	43.80	0.00
% Reads 1 MM	43.50	46.84	0.00
% Reads 2 MM	29.69	9.28	0.00
% Reads 3MM	0.22	0.08	0.00
% Reads with 3' MM	64.27	75.44	0.00
% Reads with Mid' MM	15.38	12.56	0.00
% Reads with 5' MM	20.35	12.00	0.00
Average Primer Tm	57.98	58.11	0.00
Average Shannon	2.23	1.90	0.00

Experiment: D2
Primers: 10
Templates: 4

Template Profiles

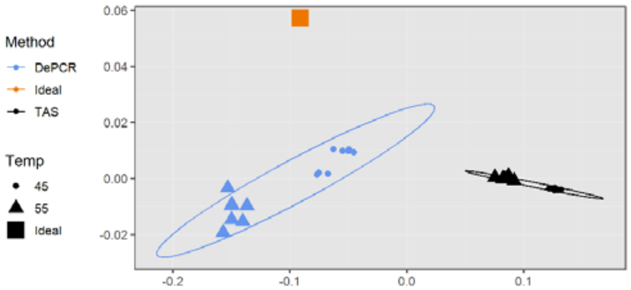

ANOSIM

DePCR vs TAS:
R=1, P=0.0001

TAS45 vs TAS55:
R=1; p=0.0002

DePCR45 vs DePCR55:
R=1 P=0.0002

	Comparison	Average Ideal Score	ANOVA
1	TAS	41.16	0.00
	DePCR	13.23	
2	TAS45	45.17	0.00
	TAS55	36.58	
3	DePCR45	9.53	0.00
	DePCR55	17.45	

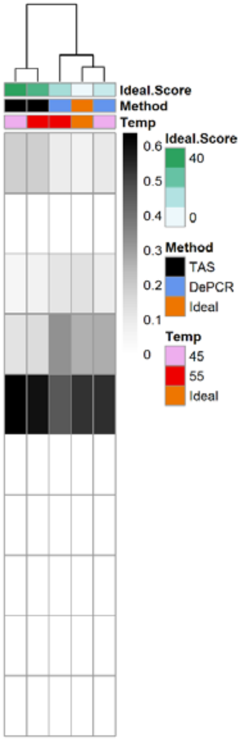

Fig. S15

Primer Utilizing Profiles

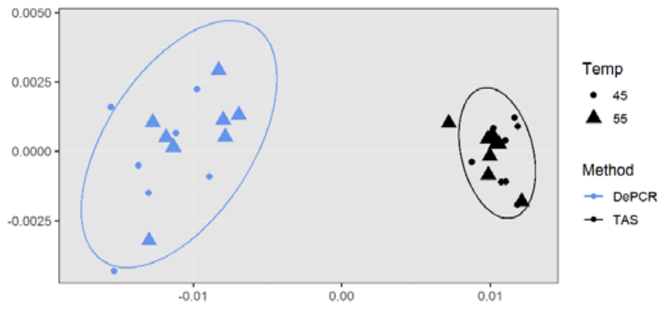

ANOSIM

DePCR vs TAS:
R=1, P=0.0001

TAS45 vs TAS55:
R=0.126; p=0.054

DePCR45 vs DePCR55:
R=0.078, P=0.174

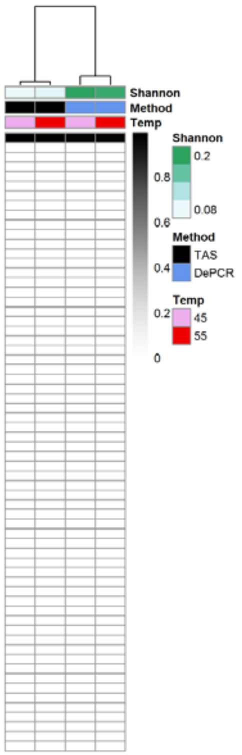

Comparison	DePCR45	DePCR55	ANOVA (p-value)
% Reads 0 MM	9.30	9.15	0.48
% Reads 1 MM	89.14	89.39	0.29
% Reads 2 MM	1.54	1.45	0.31
% Reads 3MM	0.02	0.01	0.23
% Reads with 3' MM	0.81	0.77	0.34
% Reads with Mid' MM	97.93	98.03	0.37
% Reads with 5' MM	1.26	1.20	0.54
Average Primer Tm	57.42	57.42	0.76
Average Shannon	0.20	0.19	0.10

Experiment: E1
Primers: 1
Templates: 4

Template Profiles

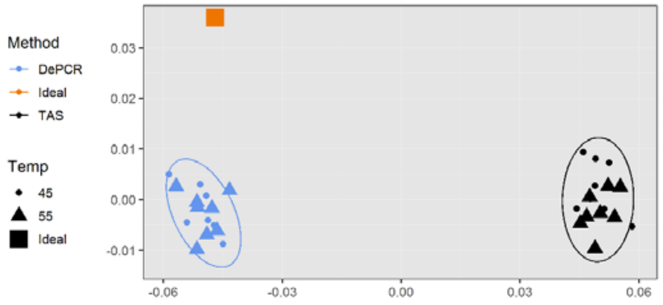

ANOSIM

DePCR vs TAS:
R=1, P=0.0001

TAS45 vs TAS55:
R=0.1; p=0.116

DePCR45 vs DePCR55:
R=-0.103, P=0.963

	Comparison	Average Ideal Score	ANOVA
1	TAS	20.53	0.00
	DePCR	7.30	
2	TAS45	20.28	0.26
	TAS55	20.78	
3	DePCR45	7.11	0.56
	DePCR55	7.47	

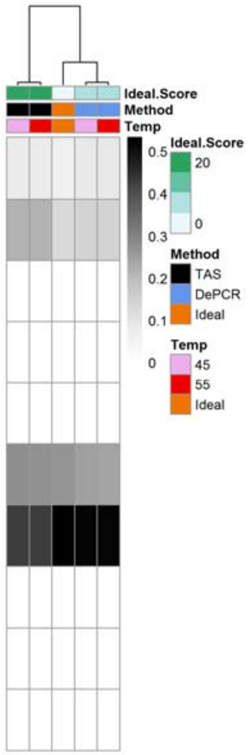

Fig. S16

Primer Utilizing Profiles

Experiment: E2
Primers: 10
Templates: 4

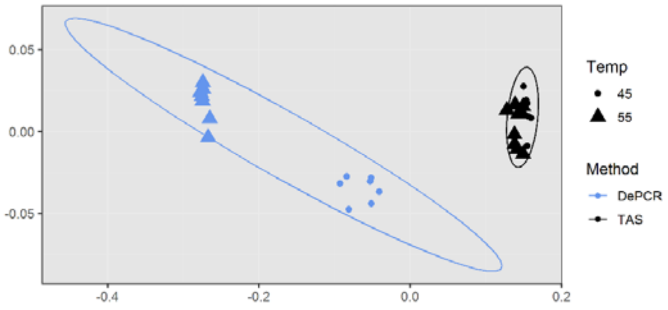

ANOSIM

DePCR vs TAS:
R=0.893, P=0.0001

TAS45 vs TAS55:
R=0.743; p=0.0002

DePCR45 vs DePCR55:
R=1, P=0.0006

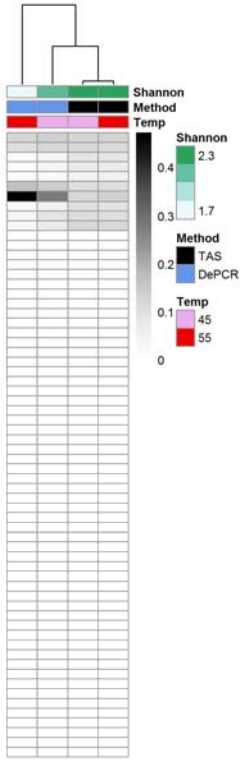

Comparison	DePCR45	DePCR55	ANOVA
**% Reads 0 MM**	30.88	51.68	0.00
**% Reads 1 MM**	41.43	38.94	0.00
**% Reads 2 MM**	27.43	9.31	0.00
**% Reads 3MM**	0.25	0.07	0.00
**% Reads with 3' MM**	10.65	7.69	0.00
**% Reads with Mid' MM**	66.09	76.16	0.00
**% Reads with 5' MM**	23.26	16.15	0.00
**Average Primer Tm**	58.44	58.93	0.00
**Average Shannon**	2.16	1.70	0.00

Template Profiles

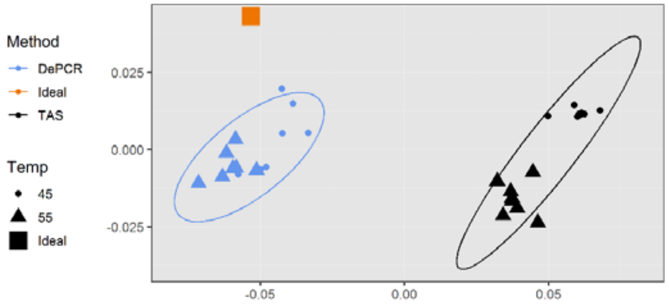

ANOSIM

DePCR vs TAS:
R=1, P=0.0001

TAS45 vs TAS55:
R=0.984; p=0.0002

DePCR45 vs DePCR55:
R=0.633, P=0.0001

	Comparison	Average Ideal Score	ANOVA
1	TAS	21.50	0.00
	DePCR	4.91	
2	TAS45	21.66	0.59
	TAS55	21.35	
3	DePCR45	5.95	0.00
	DePCR55	3.87	

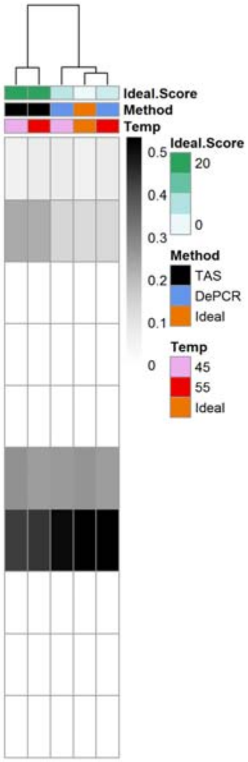

Supplement: Supplemental Information 1 [file peerj-12-17787-s001.pdf]
