# Supplementary material for: Quantitating primer-template interactions using deconstructed PCR
Source: PeerJ. 2024 Aug 8;12:e17787. doi: 10.7717/peerj.17787 (PMC11317036; doi:10.7717/peerj.17787)
Supplement: Supplemental Information 2 [file peerj-12-17787-s002.pdf]

Figures S17-18. Effect of PCR methodology and annealing temperature on template profiles in amplification reactions utilizing varying primer pools. One-way clustered heatmaps of untransformed template utilization profiling during amplification of an uneven pooling of synthetic DNA templates and varying primer pools (Figure S17 = C1, C2 and C3 experiments with all ten templates present, and template ST1 at 1/10th the concentration of the other nine templates; Figure S18 = D1, D2, E1 and E2 experiments with four templates). For experiments C1, D1 and E1, only a single primer variant was used (806F_v1), while in experiments C2, D2 and E2, 10 primers were used. In experiment C3, 9 primers were used (806F_v1 was removed). Primer and template details are shown in Table 1. Samples (columns) are color-coded by amplification method (TAS or DePCR), amplification annealing temperature (45°C or 55°C), and average Ideal score. Each column represents the average of 7-8 technical replicates per condition and rarefaction to 7,000 sequences/replicate. Templates (rows) represent all 10 templates (ordered from top to bottom; ST1, ST4, ST6, ST7, ST8, ST11, ST15, ST23, ST39, and ST55). Ideal score comparisons between TAS and DePCR (across both annealing temperatures), within TAS (45°C or 55°C), and within DePCR (45°C or 55°C) are shown in tables. Asterisks indicate significant differences in measured values by annealing temperature (ANOVA, $P < 0.01$). Intensity scales vary between experiments.

Fig. S17

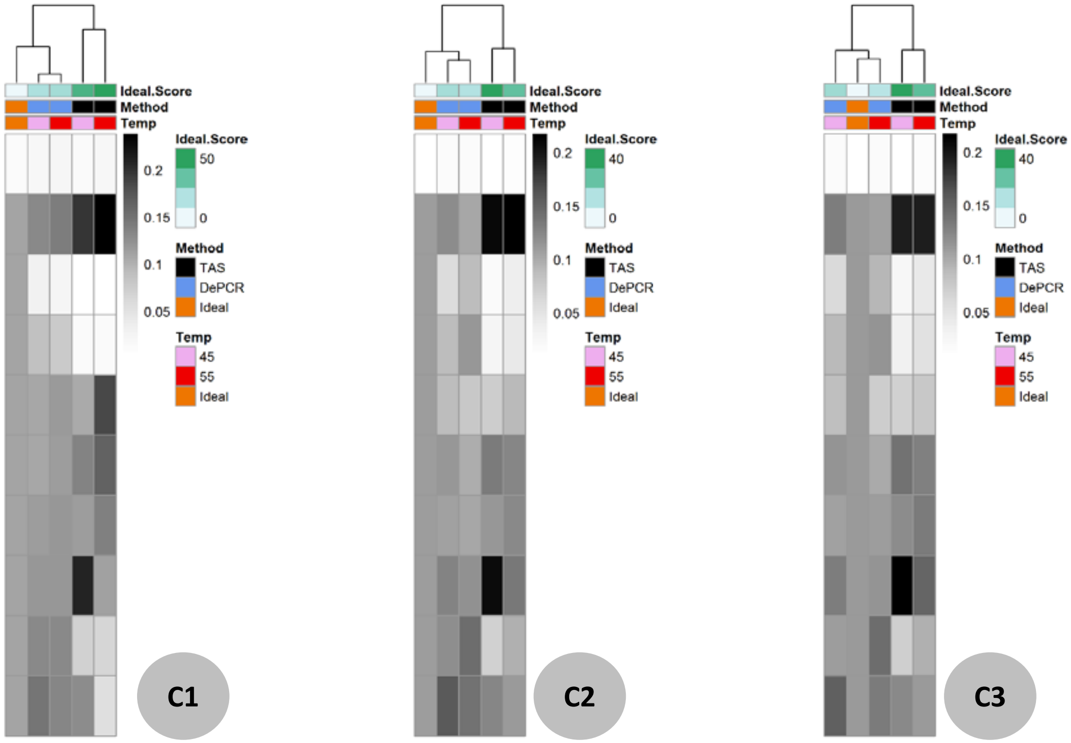

Average Ideal Score

TAS	54.32	*
DePCR	22.45	
TAS45	48.72	*
TAS55	59.91	
DePCR45	21.04	*
DePCR55	23.87	

TAS	41.48	*
DePCR	17.56	
TAS45	49.17	*
TAS55	33.79	
DePCR45	19.07	*
DePCR55	16.04	

TAS	41.53	*
DePCR	17.05	
TAS45	48.81	*
TAS55	34.24	
DePCR45	20.19	*
DePCR55	14.31	

Fig. S18

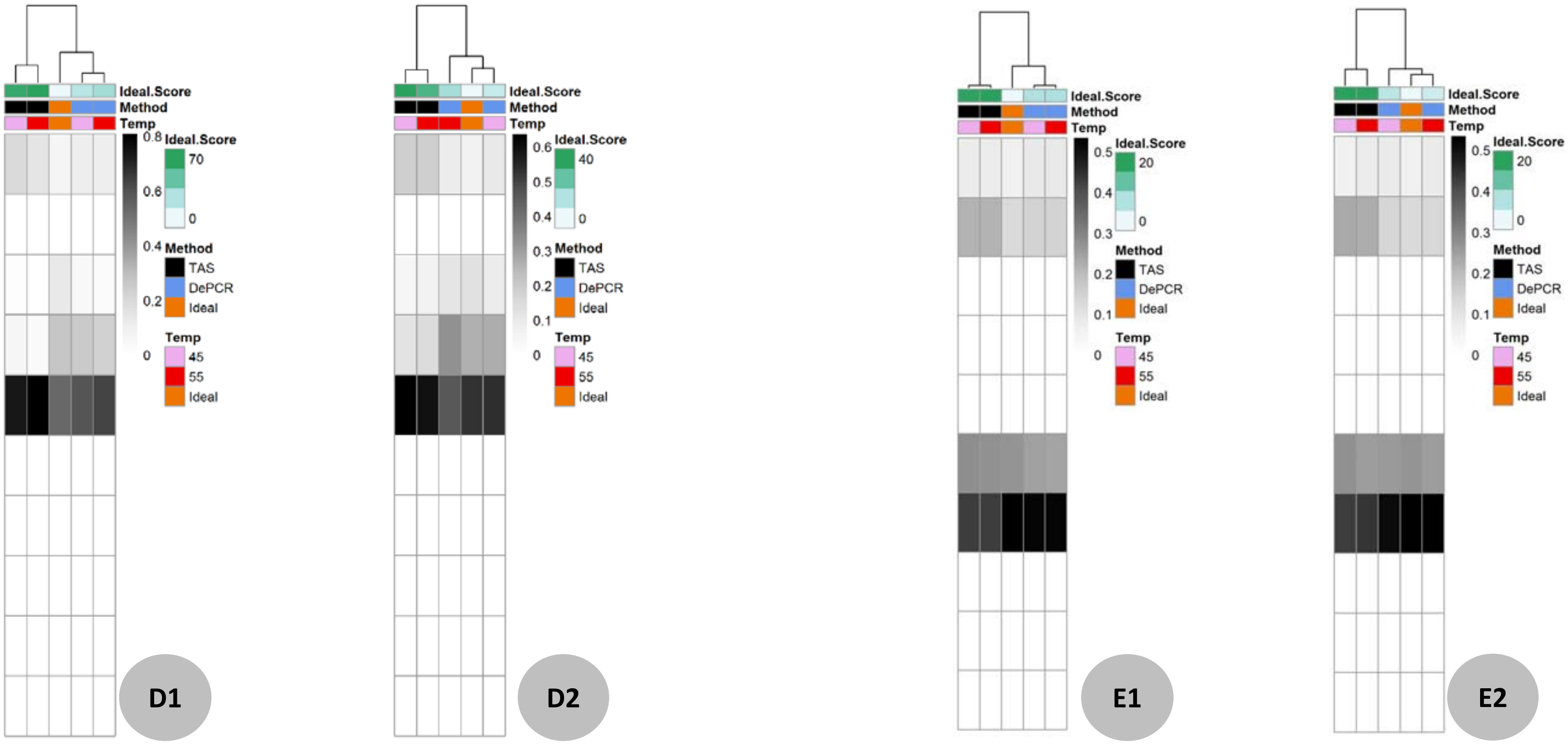

Average Ideal Score

	D1		D2	
TAS	68.46	*	41.16	*
DePCR	24.27		13.23	
TAS45	65.95	*	45.17	*
TAS55	70.96		36.58	
DePCR45	20.15	*	9.53	*
DePCR55	27.87		17.45	

Average Ideal Score

	E1		E2	
TAS	20.53	*	21.50	*
DePCR	7.30		4.91	
TAS45	20.28		21.66	
TAS55	20.78		21.35	
DePCR45	7.11		5.95	*
DePCR55	7.47		3.87	
