# Supplementary material for: Quantitating primer-template interactions using deconstructed PCR
Source: PeerJ. 2024 Aug 8;12:e17787. doi: 10.7717/peerj.17787 (PMC11317036; doi:10.7717/peerj.17787)
Supplement: Supplemental Information 9 [file peerj-12-17787-s009.docx]

**Supplemental Materials 1: Synthetic DNA template creation and sequences**

>*Rhodanobacter denitrificans* strain 2APBS1 16S ribosomal RNA gene (NR_102497)

CTGGCTCAGATTGAACGCTGGCGGCATGCCTAACACATGCAAGTCGAACGGCAGCACAGCAGTAGCAATACTGTGGGTGGCGAGTGGCGGACGGGTGAGTAATGCATCGGGATCTACCCTGACGTGGGGGATAACCTCGGGAAACCGGGACTAATACCGCATACGTCCTACGGGAGAAAGCGGGGGACCTTCGGGCCTCGCGCGGCAGGACGAACCGATGTGCGATTAGCTAGTTGGCGGGGTAATGGCCCACCAAGGCGACGATCGCTAGCTGGTCTGAGAGGATGATCAGCCACACTGGGACTGAGACACGGCCCAGACTCCTACGGGAGGCAGCAGTGGGGAATATTGGACAATGGGCGCAAGCCTGATCCAGCAATGCCGCGTGTGTGAAGAAGGCCTTCGGGTTGTAAAGCACTTTTATCAGGAGCGAAATACCACGGGTTAATACCCTATGGGGCTGACGGTACCTGAGGAATAAGCACCGGCTAACTTCGTGCCAGCAGCCGCGGTAATACGAAGGGTGCAAGCGTTAATCGGAATTACTGGGCGTAAAGGGTGCGTAGGCGGTTACTTAAGTCTGTCGTGAAATCCCCGGGCTCAACCTGGGAATGGCGATGGATACTGGGTGGCTAGAGTGTGTCAGAGGATGGTGGAATTCCCGGTGTAGCGGTGAAATGCGTAGAGATCGGGAGGAACATCAGTGGCGAAGGCGGCCATCTGGGACAACACTGACGCTGAAGCACGAAAGCGTGGGGAGCAAACAGGATTAGATACCCTGGTAGTCCACGCCCTAAACGATGCGAACTGGATGTTGGTCTCAACTCGGAGATCAGTGTCGAAGCTAACGCGTTAAGTTCGCCGCCTGGGGAGTACGGTCGCAAGACTGAAACTCAAAGGAATTGACGGGGGCCCGCACAAGCGGTGGAGTATGTGGTTTAATTCGATGCAACGCGAAGAACCTTACCTGGCCTTGACATGTCCGGAATCCTGCAGAGATGCGGGAGTGCCTTCGGGAATCGGAACACAGGTGCTGCATGGCTGTCGTCAGCTCGTGTCGTGAGATGTTGGGTTAAGTCCCGCAACGAGCGCAACCCTTGTCCTTAGTTGCCAGCACGTAATGGTGGGAACTCTAAGGAGACTGCCGGTGACAAACCGGAGGAAGGTGGGGATGACGTCAAGTCATCATGGCCCTTACGGCCAGGGCTACACACGTACTACAATGGTCGGTACAGAGGGTTGCAATACCGCGAGGTGGAGCCAATCCCAGAAAGCCGATCCCAGTCCGGATTGGAGTCTGCAACTCGACTCCATGAAGTCGGAATCGCTAGTAATCGCGGATCAGCTATGCCGCGGTGAATACGTTCCCGGGCCTTGTACACACCGCCCGTCACACCATGGGAGTGGGTTGCTCCAGAAGGCGTTAGTCTAACCGCAAGGGGGACGACGCCCACGGAGTGGTCCATGACTGGGGTGAAGTCGTAACAAGGTAGCCGTATCGGAAGGTGCGGCTGGATCACCTCCTTT

Standard 515F primer site: GTGCCAGCAGCCGCGGTAA [515F-“Parada”: GTGYCAGCMGCCGCGGTAA]

Standard 806R primer site: ATTAGATACCCTGGTAGTCC [806R-“Apprill”: GGACTACNVGGGTWTCTAAT]

>Inverse complement of *Rhodanobacter denitrificans* strain 2APBS1 16S ribosomal RNA gene (NR_102497)

AAAGGAGGTGATCCAGCCGCACCTTCCGATACGGCTACCTTGTTACGACTTCACCCCAGTCATGGACCACTCCGTGGGCGTCGTCCCCCTTGCGGTTAGACTAACGCCTTCTGGAGCAACCCACTCCCATGGTGTGACGGGCGGTGTGTACAAGGCCCGGGAACGTATTCACCGCGGCATAGCTGATCCGCGATTACTAGCGATTCCGACTTCATGGAGTCGAGTTGCAGACTCCAATCCGGACTGGGATCGGCTTTCTGGGATTGGCTCCACCTCGCGGTATTGCAACCCTCTGTACCGACCATTGTAGTACGTGTGTAGCCCTGGCCGTAAGGGCCATGATGACTTGACGTCATCCCCACCTTCCTCCGGTTTGTCACCGGCAGTCTCCTTAGAGTTCCCACCATTACGTGCTGGCAACTAAGGACAAGGGTTGCGCTCGTTGCGGGACTTAACCCAACATCTCACGACACGAGCTGACGACAGCCATGCAGCACCTGTGTTCCGATTCCCGAAGGCACTCCCGCATCTCTGCAGGATTCCGGACATGTCAAGGCCAGGTAAGGTTCTTCGCGTTGCATCGAATTAAACCACATACTCCACCGCTTGTGCGGGCCCCCGTCAATTCCTTTGAGTTTC**AGTCTTGCGACCGTACTCCCCAGGCGGCGAACTTAACGCGTTAGCTTCGACACTGATCTCCGAGTTGAGACCAACATCCAGTTCGCATCGTTTAGGGCGTGGACTACCAGGGTATCTAATCCTGTTTGCTCCCCACGCTTTCGTGCTTCAGCGTCAGTGTTGTCCCAGATGGCCGCCTTCGCCACTGATGTTCCTCCCGATCTCTACGCATTTCACCGCTACACCGGGAATTCCACCATCCTCTGACACACTCTAGCCACCCAGTATCCATCGCCATTCCCAGGTTGAGCCCGGGGATTTCACGACAGACTTAAGTAACCGCCTACGCACCCTTTACGCCCAGTAATTCCGATTAACGCTTGCACCCTTCGTATTACCGCGGCTGCTGGCACGAAGTTAGCCGGTGCTTATTCCTCAGGTACCGTCAGCCCCATAGGGTATTAACCCGTGGT**ATTTCGCTCCTGATAAAAGTGCTTTACAACCCGAAGGCCTTCTTCACACACGCGGCATTGCTGGATCAGGCTTGCGCCCATTGTCCAATATTCCCCACTGCTGCCTCCCGTAGGAGTCTGGGCCGTGTCTCAGTCCCAGTGTGGCTGATCATCCTCTCAGACCAGCTAGCGATCGTCGCCTTGGTGGGCCATTACCCCGCCAACTAGCTAATCGCACATCGGTTCGTCCTGCCGCGCGAGGCCCGAAGGTCCCCCGCTTTCTCCCGTAGGACGTATGCGGTATTAGTCCCGGTTTCCCGAGGTTATCCCCCACGTCAGGGTAGATCCCGATGCATTACTCACCCGTCCGCCACTCGCCACCCACAGTATTGCTACTGCTGTGCTGCCGTTCGACTTGCATGTGTTAGGCATGCCGCCAGCGTTCAATCTGAGCCAG

***Underlined and bold region indicates area of the gene used for synthetic gBLOCK synthesis, modified as shown below.**

**Synthetic Template Design**

AGTCTTGCGACCGTACTCCCCAGGCGGCGAACTTAACGCGTTAGCTTCGACACTGATCTCCGAGTTGAGACCAACATCCAGTTCGCATCGTTTAGGGCGTGGACTACCAGGGTATCTAATCCTGTTTGCTCCCCACGCTTTCGTGCTTCAGCGTCAGTGTTGTCCCAGATGGCCGCCTTCGCCACTGATGTTCCTCCCGATCTCTACGCATTTCACCGCTACACCGGGAATTCCACCATCCTCTGACACACTCTAGCCACCCAGTATCCATCGCCATTCCCAGGTTGAGCCCGGGGATTTCACGACAGACTTAAGTAACCGCCTACGCACCCTTTACGCCCAGTAATTCCGATTAACGCTTGCACCCTTCGTATTACCGCGGCTGCTGGCACGAAGTTAGCCGGTGCTTATTCCTCAGGTACCGTCAGCCCCATAGGGTATTAACCCGTGGT

**Step 1:** Select region of *R. denitrificans* 2APBS 16S rRNA gene surrounding the standard EMP 515F/806R primer pair which generate a 292 bp amplicon with this template. Total length of fragment is 452 bp.

**Step 2:** Use the sequence for the 806R primer as the default “forward” primer (GGACTACCAGGGTATCTAAT). The standard 806R primer for the EMP is 24-fold degenerate. We use only the variant matching *R. denitrificans* 2APBS1 for the default primer sequence (Rh806Syn_1). The primer site is shown in light blue, above. In other templates that template 1, this primer site is altered.

**Step 3**: Identify a ‘recognition’ sequence in the DNA that will be varied from template to template. The same nucleotides are used to maintain GC content, but the sequence is scrambled so that many mismatches between the recognition sequences are present. The recognition sequence for template 1 is simply a 12 base region of the original DNA from *R. denitrificans* 2APBS1 (CGATCTCTACGC), and is highlighted in red.

**Step 4:** A new reverse primer site is developed to decrease the size of the amplicon to allow for better merging with 2x153 base sequencing on the Illumina MiniSeq. No degeneracies are used here, and all synthetic templates retain this sequence. Primer modifications are only performed at the ‘806R’ primer site. The chosen primer design was: CGGAATTACTGGGCGTAAAGG (inverse complement = CCTTTACGCCCAGTAATTCCG), and the annealing location is highlighted in grey. The entire amplicon is 251 bp in size.

>ST1 (new ST0)
AGTCTTGCGACCGTACTCCCCAGGCGGCGAACTTAACGCGTTAGCTTCGACACTGATCTCCGAGTTGAGACCAACATCCAGTTCGCATCGTTTAGGGCGTGGACTACCAGGGTATCTAATCCTGTTTGCTCCCCACGCTTTCGTGCTTCAGCGTCAGTGTTGTCCCAGATGGCCGCCTTCGCCACTGATGTTCCTCCCGATCTCTACGCATTTCACCGCTACACCGGGAATTCCACCATCCTCTGACACACTCTAGCCACCCAGTATCCATCGCCATTCCCAGGTTGAGCCCGGGGATTTCACGACAGACTTAAGTAACCGCCTACGCACCCTTTACGCCCAGTAATTCCGATTAACGCTTGCACCCTTCGTATTACCGCGGCTGCTGGCACGAAGTTAGCCGGTGCTTATTCCTCAGGTACCGTCAGCCCCATAGGGTATTAACCCGTGGT

Rh806 variant: GGACTACCAGGGTATCTAAT
Recognition sequence: CGATCTCTACGC
Rh555 sequence: CGGAATTACTGGGCGTAAAGG (IC: CCTTTACGCCCAGTAATTCCG)

>ST4 (new ST5)
AGTCTTGCGACCGTACTCCCCAGGCGGCGAACTTAACGCGTTAGCTTCGACACTGATCTCCGAGTTGAGACCAACATCCAGTTCGCATCGTTTAGGGCGTGGACTACCAGGGCATCTAATCCTGTTTGCTCCCCACGCTTTCGTGCTTCAGCGTCAGTGTTGTCCCAGATGGCCGCCTTCGCCACTGATGTTCCTCCTGCGCTCCAACTATTTCACCGCTACACCGGGAATTCCACCATCCTCTGACACACTCTAGCCACCCAGTATCCATCGCCATTCCCAGGTTGAGCCCGGGGATTTCACGACAGACTTAAGTAACCGCCTACGCACCCTTTACGCCCAGTAATTCCGATTAACGCTTGCACCCTTCGTATTACCGCGGCTGCTGGCACGAAGTTAGCCGGTGCTTATTCCTCAGGTACCGTCAGCCCCATAGGGTATTAACCCGTGGT

Rh806 variant: GGACTACCAGGGCATCTAAT
Recognition sequence: TGCGCTCCAACT
Rh555 sequence: CGGAATTACTGGGCGTAAAGG (IC: CCTTTACGCCCAGTAATTCCG)

>ST6 (new ST7)
AGTCTTGCGACCGTACTCCCCAGGCGGCGAACTTAACGCGTTAGCTTCGACACTGATCTCCGAGTTGAGACCAACATCCAGTTCGCATCGTTTAGGGCGTGGACTACCAGGGTATCTACTCCTGTTTGCTCCCCACGCTTTCGTGCTTCAGCGTCAGTGTTGTCCCAGATGGCCGCCTTCGCCACTGATGTTCCTCCCTTAGCATGCCCATTTCACCGCTACACCGGGAATTCCACCATCCTCTGACACACTCTAGCCACCCAGTATCCATCGCCATTCCCAGGTTGAGCCCGGGGATTTCACGACAGACTTAAGTAACCGCCTACGCACCCTTTACGCCCAGTAATTCCGATTAACGCTTGCACCCTTCGTATTACCGCGGCTGCTGGCACGAAGTTAGCCGGTGCTTATTCCTCAGGTACCGTCAGCCCCATAGGGTATTAACCCGTGGT

Rh806 variant: GGACTACCAGGGTATCTACT
Recognition sequence: CTTAGCATGCCC
Rh555 sequence: CGGAATTACTGGGCGTAAAGG (IC: CCTTTACGCCCAGTAATTCCG)

>ST7 (new ST9)
AGTCTTGCGACCGTACTCCCCAGGCGGCGAACTTAACGCGTTAGCTTCGACACTGATCTCCGAGTTGAGACCAACATCCAGTTCGCATCGTTTAGGGCGTGGACTACCAGGGTATCTATTCCTGTTTGCTCCCCACGCTTTCGTGCTTCAGCGTCAGTGTTGTCCCAGATGGCCGCCTTCGCCACTGATGTTCCTCCCGTCCATCTACGATTTCACCGCTACACCGGGAATTCCACCATCCTCTGACACACTCTAGCCACCCAGTATCCATCGCCATTCCCAGGTTGAGCCCGGGGATTTCACGACAGACTTAAGTAACCGCCTACGCACCCTTTACGCCCAGTAATTCCGATTAACGCTTGCACCCTTCGTATTACCGCGGCTGCTGGCACGAAGTTAGCCGGTGCTTATTCCTCAGGTACCGTCAGCCCCATAGGGTATTAACCCGTGGT

Rh806 variant: GGACTACCAGGGTATCTATT
Recognition sequence: CGTCCATCTACG
Rh555 sequence: CGGAATTACTGGGCGTAAAGG (IC: CCTTTACGCCCAGTAATTCCG)

>ST8 (new ST8)
AGTCTTGCGACCGTACTCCCCAGGCGGCGAACTTAACGCGTTAGCTTCGACACTGATCTCCGAGTTGAGACCAACATCCAGTTCGCATCGTTTAGGGCGTGGACTACCAGGGTATCTAGTCCTGTTTGCTCCCCACGCTTTCGTGCTTCAGCGTCAGTGTTGTCCCAGATGGCCGCCTTCGCCACTGATGTTCCTCCTCTACCGATGCCATTTCACCGCTACACCGGGAATTCCACCATCCTCTGACACACTCTAGCCACCCAGTATCCATCGCCATTCCCAGGTTGAGCCCGGGGATTTCACGACAGACTTAAGTAACCGCCTACGCACCCTTTACGCCCAGTAATTCCGATTAACGCTTGCACCCTTCGTATTACCGCGGCTGCTGGCACGAAGTTAGCCGGTGCTTATTCCTCAGGTACCGTCAGCCCCATAGGGTATTAACCCGTGGT

Rh806 variant: GGACTACCAGGGTATCTAGT
Recognition sequence: TCTACCGATGCC
Rh555 sequence: CGGAATTACTGGGCGTAAAGG (IC: CCTTTACGCCCAGTAATTCCG)

>ST11 (new ST4)
AGTCTTGCGACCGTACTCCCCAGGCGGCGAACTTAACGCGTTAGCTTCGACACTGATCTCCGAGTTGAGACCAACATCCAGTTCGCATCGTTTAGGGCGTGGACTACCAGGGAATCTAATCCTGTTTGCTCCCCACGCTTTCGTGCTTCAGCGTCAGTGTTGTCCCAGATGGCCGCCTTCGCCACTGATGTTCCTCCGAACCTTTCCCGATTTCACCGCTACACCGGGAATTCCACCATCCTCTGACACACTCTAGCCACCCAGTATCCATCGCCATTCCCAGGTTGAGCCCGGGGATTTCACGACAGACTTAAGTAACCGCCTACGCACCCTTTACGCCCAGTAATTCCGATTAACGCTTGCACCCTTCGTATTACCGCGGCTGCTGGCACGAAGTTAGCCGGTGCTTATTCCTCAGGTACCGTCAGCCCCATAGGGTATTAACCCGTGGT

Rh806 variant: GGACTACCAGGGAATCTAAT
Recognition sequence: GAACCTTTCCCG
Rh555 sequence: CGGAATTACTGGGCGTAAAGG (IC: CCTTTACGCCCAGTAATTCCG)

>ST15 (new ST6)
AGTCTTGCGACCGTACTCCCCAGGCGGCGAACTTAACGCGTTAGCTTCGACACTGATCTCCGAGTTGAGACCAACATCCAGTTCGCATCGTTTAGGGCGTGGACTACCAGGGGATCTAATCCTGTTTGCTCCCCACGCTTTCGTGCTTCAGCGTCAGTGTTGTCCCAGATGGCCGCCTTCGCCACTGATGTTCCTCCGACCCTAGCTTCATTTCACCGCTACACCGGGAATTCCACCATCCTCTGACACACTCTAGCCACCCAGTATCCATCGCCATTCCCAGGTTGAGCCCGGGGATTTCACGACAGACTTAAGTAACCGCCTACGCACCCTTTACGCCCAGTAATTCCGATTAACGCTTGCACCCTTCGTATTACCGCGGCTGCTGGCACGAAGTTAGCCGGTGCTTATTCCTCAGGTACCGTCAGCCCCATAGGGTATTAACCCGTGGT

Rh806 variant: GGACTACCAGGGGATCTAAT
Recognition sequence: GACCCTAGCTTC
Rh555 sequence: CGGAATTACTGGGCGTAAAGG (IC: CCTTTACGCCCAGTAATTCCG)

>ST23 (new ST2)
AGTCTTGCGACCGTACTCCCCAGGCGGCGAACTTAACGCGTTAGCTTCGACACTGATCTCCGAGTTGAGACCAACATCCAGTTCGCATCGTTTAGGGCGTGGACTATCAGGGTATCTAATCCTGTTTGCTCCCCACGCTTTCGTGCTTCAGCGTCAGTGTTGTCCCAGATGGCCGCCTTCGCCACTGATGTTCCTCCTGCCAGCCCTATATTTCACCGCTACACCGGGAATTCCACCATCCTCTGACACACTCTAGCCACCCAGTATCCATCGCCATTCCCAGGTTGAGCCCGGGGATTTCACGACAGACTTAAGTAACCGCCTACGCACCCTTTACGCCCAGTAATTCCGATTAACGCTTGCACCCTTCGTATTACCGCGGCTGCTGGCACGAAGTTAGCCGGTGCTTATTCCTCAGGTACCGTCAGCCCCATAGGGTATTAACCCGTGGT

Rh806 variant: GGACTATCAGGGTATCTAAT
Recognition sequence: TGCCAGCCCTAT
Rh555 sequence: CGGAATTACTGGGCGTAAAGG (IC: CCTTTACGCCCAGTAATTCCG)

>ST39 (new ST3)
AGTCTTGCGACCGTACTCCCCAGGCGGCGAACTTAACGCGTTAGCTTCGACACTGATCTCCGAGTTGAGACCAACATCCAGTTCGCATCGTTTAGGGCGTGGACTAACAGGGTATCTAATCCTGTTTGCTCCCCACGCTTTCGTGCTTCAGCGTCAGTGTTGTCCCAGATGGCCGCCTTCGCCACTGATGTTCCTCCACATCGCTCGTCATTTCACCGCTACACCGGGAATTCCACCATCCTCTGACACACTCTAGCCACCCAGTATCCATCGCCATTCCCAGGTTGAGCCCGGGGATTTCACGACAGACTTAAGTAACCGCCTACGCACCCTTTACGCCCAGTAATTCCGATTAACGCTTGCACCCTTCGTATTACCGCGGCTGCTGGCACGAAGTTAGCCGGTGCTTATTCCTCAGGTACCGTCAGCCCCATAGGGTATTAACCCGTGGT

Rh806 variant: GGACTAACAGGGTATCTAAT
Recognition sequence: ACATCGCTCGTC
Rh555 sequence: CGGAATTACTGGGCGTAAAGG (IC: CCTTTACGCCCAGTAATTCCG)

>ST55 (new ST1)
AGTCTTGCGACCGTACTCCCCAGGCGGCGAACTTAACGCGTTAGCTTCGACACTGATCTCCGAGTTGAGACCAACATCCAGTTCGCATCGTTTAGGGCGTGGACTAGCAGGGTATCTAATCCTGTTTGCTCCCCACGCTTTCGTGCTTCAGCGTCAGTGTTGTCCCAGATGGCCGCCTTCGCCACTGATGTTCCTCCCTTACCAGTCGCATTTCACCGCTACACCGGGAATTCCACCATCCTCTGACACACTCTAGCCACCCAGTATCCATCGCCATTCCCAGGTTGAGCCCGGGGATTTCACGACAGACTTAAGTAACCGCCTACGCACCCTTTACGCCCAGTAATTCCGATTAACGCTTGCACCCTTCGTATTACCGCGGCTGCTGGCACGAAGTTAGCCGGTGCTTATTCCTCAGGTACCGTCAGCCCCATAGGGTATTAACCCGTGGT

Rh806 variant: GGACTAGCAGGGTATCTAAT
Recognition sequence: CTTACCAGTCGC
Rh555 sequence: CGGAATTACTGGGCGTAAAGG (IC: CCTTTACGCCCAGTAATTCCG)
