# Supplementary material for: Quantitating primer-template interactions using deconstructed PCR
Source: PeerJ. 2024 Aug 8;12:e17787. doi: 10.7717/peerj.17787 (PMC11317036; doi:10.7717/peerj.17787)
Supplement: Supplemental Information 11 [file peerj-12-17787-s011.zip › Supplemental_Material_3/Readme.docx]

R.Scripts

It is expected that all files and folders will be placed in a project folder.

Variable project_folder in the scripts should be set that.

The wd variable should be modified to be the working directory.

At the beginning of scripts there are options to start from the beginning (fastq files), if you want to skip that I included the initial data_tables created from the fastq files.

If you want to work from fastq files you can download them from the Sequence Read Archive (SRA) of the National Center for Biotechnology Information (NCBI) BioProject identifiers PRJNA513137 and PRJNA1072695.

The fastq files would need to be merged and renamed appropriately. There are some additional resources in the stuff folder that may be of use. However, I only downloaded the files from the initial experiments.
